# Host Adaptability and Genetic Mechanisms of the Rice Strain of Fall Armyworm (*Spodoptera frugiperda*)

**DOI:** 10.3390/insects16101029

**Published:** 2025-10-06

**Authors:** Hanyue Wang, Chao Wu, Kenneth Wilson, Yutao Xiao, Kaiyu Liu

**Affiliations:** 1 Key Laboratory of Pesticide & Chemical Biology of Ministry of Education, Hubei Key Laboratory of Genetic Regulation and Integrative Biology, School of Life Sciences, Central China Normal University, Wuhan 430079, China; wanghanyue@mails.ccnu.edu.cn; 2 Lancaster Environment Centre, Lancaster University, Lancaster LA1 4YQ, UK; wuchao@caas.cn (C.W.); ken.wilson@lancaster.ac.uk (K.W.); 3 Shenzhen Branch, Guangdong Laboratory of Lingnan Modern Agriculture, Key Laboratory of Gene Editing Technologies (Hainan), Ministry of Agriculture and Rural Affairs, Agricultural Genomics Institute at Shenzhen, Chinese Academy of Agricultural Sciences, Shenzhen 518120, China

**Keywords:** fall armyworm, rice strain, host adaptability, genetic mechanisms

## Abstract

The fall armyworm (*Spodoptera frugiperda*) is an invasive pest of global concern, posing a significant threat to food security. It can be divided into two biotypes: the rice strain (RS) and the corn strain (CS). These two biotypes are nearly indistinguishable morphologically but differ significantly in host adaptability. The two biotypes can hybridize, but the efficiency of their hybridization has not been conclusively determined. Our research has found that the two biotypes of fall armyworm exhibit significant differences in weight gain when feeding on different host plants and artificial diets. The rice strain has a broader diet range, showing notably stronger adaptability to rice and ryegrass compared to the corn strain, suggesting that the rice strain may possess more robust detoxification metabolism. Under laboratory conditions, the two biotypes can hybridize effectively, and their offspring exhibit certain hybrid advantages. The host adaptability traits of the rice strain are dominantly inherited. Our findings provide an important foundation for understanding the biological basis of host adaptability in the rice strain of fall armyworm.

## Simple Summary

The fall armyworm is a major invasive pest that threatens global food security. It consists of two nearly identical biotypes—the rice strain (RS) and corn strain (CS)—which differ in host plant adaptation. Laboratory assays shows that the RS has a wider host range and stronger detoxification ability, especially on rice and ryegrass, compared to the CS. Although they can interbreed, hybridization outcomes are complex. Hybrid offspring display advantages in certain traits, with RS-like host adaptability being dominant. These results help clarify the genetic mechanisms behind RS’s broader adaptability.

## 1. Introduction

The fall armyworm (*Spodoptera frugiperda* J. E. Smith), a highly migratory and polyphagous pest native to subtropical and tropical regions of the Americas (order Lepidoptera, family Noctuidae), poses a significant global threat to agriculture [1,2]. Since its spread beyond its native range, it has caused extensive damage to key crops such as corn, cotton, and legumes across the Americas, Africa, and Asia [3,4,5,6].

In its native range, *S. frugiperda* comprises two genetically distinct but morphologically similar biotypes: the rice strain (RS) and corn strain (CS) [7,8]. While both strains exhibit overlapping host ranges, they display divergent ecological adaptations in host preference, oviposition behavior, mating strategies, and environmental tolerance [9,10,11,12]. The CS predominantly infests corn, sorghum, sugarcane, and cotton, whereas the RS shows broader polyphagy, favoring rice, alfalfa, millet, and grasses [13]. However, this differentiation is not absolute; the RS demonstrates considerable plasticity, often thriving on CS-preferred hosts like corn [14], blurring the boundaries between biotype-specific adaptations.

This ecological overlap raises critical questions about the drivers of their differentiation and the potential for host shifts—particularly in invasive contexts. A key knowledge gap persists regarding whether the RS retains inherent adaptability to rice in regions where rice cultivation is limited, which could predict its invasive risk to Asian rice systems. Moreover, the palatability of rice to both biotypes remains understudied, despite its implications for food security in the Eastern Hemisphere. Resolving these uncertainties is urgent, as climate change and agricultural intensification may accelerate host range expansions, amplifying the pest’s global impact.

Understanding the mechanisms underlying biotype divergence requires examining both ecological specialization and reproductive barriers. From an ecological physiology perspective, Hay-Roe et al. demonstrated that the RS exhibits broader host acceptance than the CS, particularly on *Cynodon* grasses, suggesting adaptive divergence in metabolic flexibility and detoxification capacity [15]. Evolutionary studies by Pashley and Martin further revealed asymmetric reproductive isolation, with female CS × male RS crosses showing reduced compatibility—highlighting how reproductive barriers may help maintain biotype divergence [8]. However, the proximate physiological mechanisms and ultimate evolutionary drivers of this divergence remain incompletely characterized.

Hybridization between closely related species or subspecies can provide insights into genetic differentiation and reproductive compatibility. For example, hybridization between *Helicoverpa armigera* and *H. assulta* is highly incompatible, making fertile offspring rare [16]. Similarly, *Spodoptera litura* and *S. littoralis* exhibit hybridization incompatibility [17]. In *S. frugiperda*, reports on hybridization efficiency between the two biotypes have been inconsistent [18]. Understanding these dynamics is critical for pest management, as hybridization barriers influence gene flow and evolutionary trajectories. Conversely, successful hybridization could lead to novel hybrid populations with altered host preferences, pesticide resistance, or expanded geographic ranges—complicating pest control strategies.

To systematically address these knowledge gaps, we formulated three testable hypotheses:

(1) Host Range Hypothesis: The RS possesses superior dietary adaptability compared to the CS, particularly manifesting as enhanced growth performance on non-preferred hosts (rice and ryegrass), attributable to more efficient detoxification metabolic pathways.
(2) Specialization Hypothesis: The CS demonstrates optimal growth and development specifically on its preferred host (corn), with significantly reduced performance on alternative hosts, reflecting evolutionary specialization and narrower physiological adaptation.
(3) Hybridization Hypothesis: Contrary to previous reports of reproductive incompatibility, the two biotypes retain the capacity for successful hybridization under controlled conditions, with hybrid offspring exhibiting transgressive traits and dominant inheritance of RS-like host adaptability characteristics.

By testing these hypotheses, this study aims to clarify the ecological and evolutionary mechanisms driving biotype divergence in *S. frugiperda*, with implications for predicting its invasive potential and improving pest management strategies.

## 2. Materials and Methods

### 2.1. Insect Collection and Rearing Conditions

The experimental colonies comprised rice strain (RS) and corn strain (CS) fall armyworms. The RS colony came from pastures (mixed grasses, mostly *Cynodon* spp.) located in Franklin Parish, LA, USA near the town of Winnsboro. Larvae were collected from the field in early to mid-July 2021, and 150 larvae were sent to the USDA-ARS lab in Gainesville, FL in late July 2021. The CS colony was initiated from a commercial culture sourced from AgBiTech (Fort Worth, TX, USA). This culture was derived from field populations in the Texas region. The number of individuals obtained from the supplier was sufficient to establish a robust breeding population without introducing founder effects.

Both colonies were maintained under standardized laboratory conditions. Larvae were reared in white rearing trays (RT32W, Frontier Agricultural Sciences, Newark, DE, USA) containing fall armyworm diet (Southland Products, Lake Village, AR, USA). Neonates were individually added to the cells using a camel hair’s brush, and cells were covered with gas-permeable adhesive lids (RTCV4, Frontier Agricultural Sciences, Newark, DE, USA). After about 23 days, pupae were removed from the trays and sexed, and emerged adults were placed in cylindrical screen cages (28 cm height, 21 cm diameter). Adults were supplied with a 20% honey–sugar solution for nourishment, and paper towels (Bounty^®^, Proctor & Gamble, Cincinnati, OH, USA) were stretched at the tops of the cages as an oviposition substrate. Rearing trays and adult oviposition cages were placed in large rearing rooms under ≈23 °C, 70% RH, and 14:10 h photoperiod.

### 2.2. Biotype Analysis

The DNA extraction and biotype identification were performed as follows: Initially, one leg from each adult used for generational mating was selected for DNA extraction and detection to ensure the accuracy of their biotype. The genomic DNA was extracted using the Multisource Genomic DNA Miniprep Kit (Axygen Biosciences, Union City, CA, USA). The extracted DNA was quality-checked by 1% agarose gel electrophoresis, and its concentration was measured using a NanoDrop ND-2000 spectrophotometer (Thermo Fisher Scientific, Waltham, MA, USA). Based on published primer sequences and PCR amplification conditions [19], specific amplification of the mitochondrial *COI* gene and the nuclear *Tpi* gene fragment was performed. The PCR products were verified by 1% agarose gel electrophoresis and subsequently sequenced by Sangon Biotech (Shanghai) Co., Ltd., Shanghai, China. Finally, the biotype of the tested insects was accurately determined by comparing the specific nucleotide differences in the *COI* and *Tpi* genes between rice-strain and corn-strain fall armyworm [20,21].

### 2.3. Host Preference Bioassay

Neonate larvae from both strains were individually introduced into rearing containers at a density of 40 larvae per 10 cm^3^. Larvae were provided with fresh leaves of rice (*Oryza sativa*, variety Meixiangzhan 2), corn (*Zea mays*), ryegrass (*Lolium perenne*), or an artificial diet [22]. The rice plants were supplied by the Agricultural Genomics Institute at Shenzhen, Chinese Academy of Agricultural Sciences and grown in controlled-environment growth chambers at Lancaster Environment Centre, Lancaster University, under standard indoor cultivation conditions (27 ± 1 °C, 70 ± 5% RH). Fresh corn leaves were obtained from greenhouse-grown seedlings of the same varieties found at the original collection sites, with plants maintained under natural light conditions. Ryegrass was collected from campus lawns of Lancaster University. Larval growth parameters were quantified after 7 days of feeding.

### 2.4. Mating Success Rates

We performed reciprocal single-pair crosses between the RS and CS biotypes, including both parental strains and their hybrid offspring. Specifically, we examined RS females crossed with CS males, designated as R♀ × C♂, and CS females crossed with RS males, designated as C♀ × R♂. For each mating pair, we carefully recorded whether oviposition occurred and whether any eggs successfully hatched. Using data from multiple biological replicates, we conducted statistical comparisons of reproductive performance. Key parameters analyzed included oviposition success rate, defined as the proportion of mating pairs that successfully laid eggs, and hatching success rate, defined as the proportion of mating pairs that produced at least one hatched egg (see below).

### 2.5. Hybridization and Feeding Preference

Two hybrid lines were generated through controlled single-pair crosses: RS females × CS males (RC hybrids) and CS females × RS males (CR hybrids). Upon eclosion, F1 progeny were collected and transferred to standardized rearing containers at a density of 40 larvae per 10 cm^3^. Each container was provisioned with either fresh rice leaves or perennial ryegrass, depending on the experimental treatment. Larval growth performance was evaluated by measuring body weight following a 7-day feeding period under standard rearing conditions.

### 2.6. Statistical Analysis

All data analyses were performed using R statistical software (v4.4.0). The following packages were employed for specific analytical tasks: dplyr (v1.1.4) for data wrangling, tidyr (v1.3.1) for data tidying, ggplot2 (v3.5.1) for data visualization, and broom (v1.0.7) for statistical model output tidying. The choice of statistical tests was determined by the data type and experimental design. Continuous data (e.g., larval weight) were analyzed using analysis of variance (ANOVA) or *t*-tests, while proportional data (e.g., success rates) were analyzed using generalized linear models (GLM) with a binomial distribution. Post hoc comparisons were performed using Tukey’s HSD test following significant ANOVA results. Statistical significance was evaluated at α = 0.05 for all hypothesis tests.

## 3. Results

### 3.1. Growth and Development Rates of Rice Strain (RS) and Corn Strain (CS) Fall Armyworm on Different Host Plants

**Figure 1:**
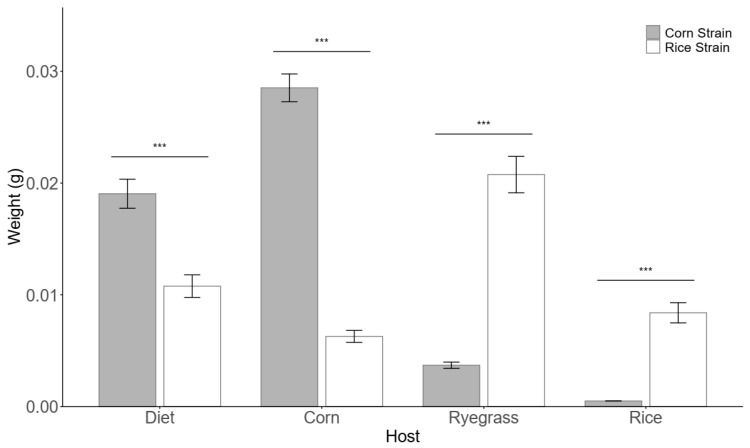
The weight (mean ± SE) of two biotypes feeding on four different foods for 7 days. Note: The dark shade indicates the corn strain, while the light shade denotes the rice strain; *** *p* ≤ 0.001.

In the Americas, the RS is often found on turfgrass, while the CS primarily inhabits corn plants, suggesting that turfgrass and corn may be the optimal host plants for the two strains, respectively. We selected corn, ryegrass (a common turfgrass species), rice, and an artificial diet to test growth and development. The results showed that after 7 days of feeding, the average weights of RS neonate larvae on rice, ryegrass, corn, and the artificial diet were 0.0084 g, 0.0208 g, 0.0063 g, and 0.0108 g, respectively. In contrast, the average weights of CS neonate larvae on the same diets were 0.0005 g, 0.0037 g, 0.0285 g, and 0.019 g, respectively. Statistical analysis revealed that the effect of host plant on larval weight was highly significant for both strains (*p* < 0.001 (Figure 1)). The RS exhibited the largest weight gain on ryegrass, with slower yet stable growth on other diets, indicating broad host adaptability. The CS showed the fastest weight gain on corn, followed by the artificial diet, ryegrass, and rice. The weight gain of CS varied significantly across hosts, with a 10-fold difference between the highest (corn) and the lowest (rice). Notably, the weight gain of CS on its optimal host (corn) was significantly greater than that of RS on its optimal host (ryegrass).

### 3.2. Interstrain and Intrastrain Mating Success Rates of RS and CS Strains

**Figure 2:**
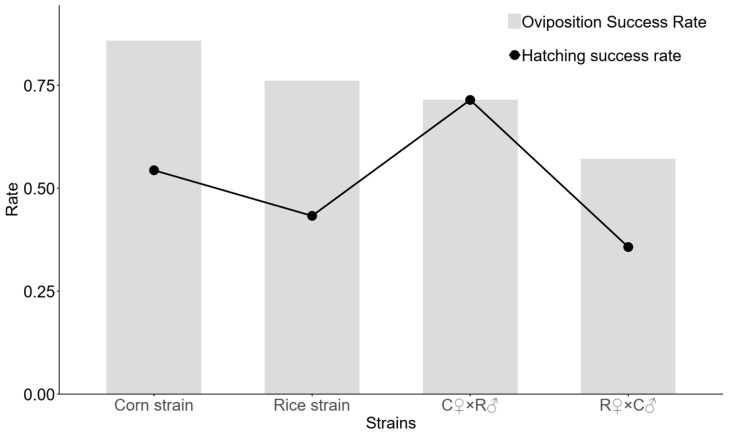
Interstrain and intrastrain mating success rates of RS and CS moth strains. Note: C♀ × R♂, Corn strain females × Rice strain males; R♀ × C♂, Rice strain females × Corn strain males.

We evaluated mating success rates both within and between the RS and CS strains, with their hybrid offspring designated as RC (from RS female × CS male crosses) and CR (from CS female × RS male crosses), respectively. The results revealed that intra-strain RS pairs achieved an oviposition success rate of 76.12% and a hatching success rate of 43.28%, while intra-strain CS pairs showed rates of 85.87% and 54.35% for oviposition and hatching, respectively, with no significant differences between strains. In inter-strain crosses, RS females paired with CS males (producing RC offspring) demonstrated success rates of 57.14% for oviposition and 35.71% for hatching. Conversely, CS females crossed with RS males (yielding CR offspring) showed higher success rates of 71.43% for both oviposition and hatching (Figure 2).

### 3.3. Inheritance of Detoxification Metabolism in the Rice Strain

**Figure 3:**
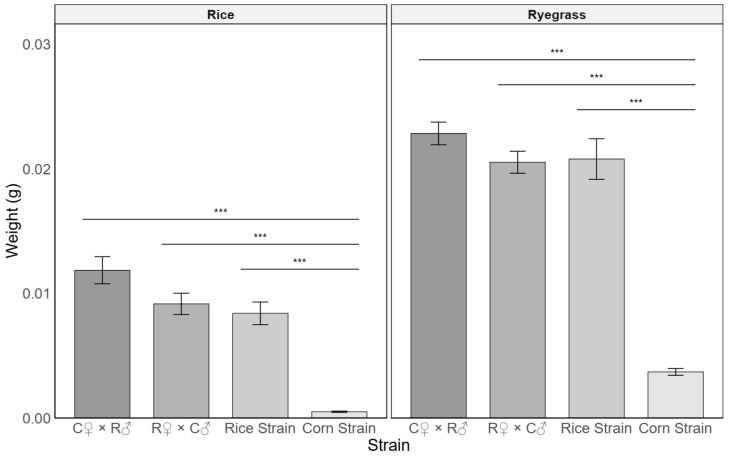
The weight (mean ± SE) of two hybrid offspring and two biotypes after 7 days of feeding on rice or ryegrass. Note: C♀ × R♂, Corn strain females × Rice strain males; R♀ × C♂, Rice strain females × Corn strain males; *** *p* ≤ 0.001.

The RS from Louisiana demonstrates broader host adaptability in this experimental setting, which is likely associated with detoxification metabolism. To preliminarily investigate the inheritance of this trait, we conducted reciprocal cross-experiments using this specific strain. The results showed that F1 offspring from both RS (female) × CS (male) and CS (female) × RS (male) crosses grew rapidly on ryegrass, exhibiting phenotypes similar to or even better than the parental RS strain. Overall, a significant difference in larval weight was found among the corn strain and hybrid groups (*p* < 0.001) (Figure 3). Notably, the F1 offspring from CS (female) × RS (male) crosses performed particularly well, with a weight significantly greater than that of the CS parent (*p* < 0.001), suggesting that under these experimental conditions, the genetic factors underlying detoxification metabolism in our RS colony appear to follow an autosomal dominant pattern, with hybrid vigor potentially contribute to the enhanced phenotype. Furthermore, the superior performance of the CR hybrid (CS female × RS male) suggests that maternal inheritance, potentially including cytoplasmic factors or egg provisioning from the CS parent, may confer additional fitness advantages to the offspring under these experimental conditions.

## 4. Discussion

The host plant specificity of the RS and CS reflects their distinct nutritional adaptations. Studies have shown that the RS exhibits slower weight gain, lower pupal weight, and reduced survival rates when feeding on corn compared to grasses. Similarly, the CS shows lower pupation rates, pupal weight, emergence rates, and fecundity when feeding on rice compared to corn [23,24]. Our findings align with these observations, but we detected even more pronounced differences in weight gain, likely due to the specific timing of our measurements. Fall armyworm larvae typically reach the third instar around 7 days after hatching, and their detoxification metabolism is weaker before this stage, making weight gain a sensitive indicator of detoxification capacity. Older larvae generally have stronger detoxification abilities, making differences less pronounced at later stages [25,26].

Research has also shown that, under laboratory conditions, the CS begins mating approximately 3 h earlier than the RS, with a broader mating time window [9]. Studies in Texas and Florida, USA, suggest these differences exist in the field [27,28]. This divergence in circadian rhythms is linked to lower expression levels of the *vrille* gene, which regulates the biological clock, in the CS. Additionally, differences in female sex pheromone composition contribute to mating behavior differences [29]. Analysis of pheromone gland extracts from female fall armyworms collected in Florida revealed that the CS contains significantly lower levels of (Z)-7-dodecenyl acetate (Z7-12: OAc) and (Z)-9-dodecenyl acetate (Z9-12: OAc) compared to the RS. Behavioral assays showed that 2% Z7-12: OAc is more attractive to CS males, while 2–10% Z7-12: OAc attracts RS males [30,31]. Field studies comparing two SNPs found that mating frequencies between the two biotypes were 4–5 times lower than those within the same biotype [32]. In contrast, our laboratory studies found no mating barriers between the two biotypes under these controlled conditions, with hybridization efficiency approaching intra-strain mating efficiency when CS females were paired with RS males. This indicates that, in the absence of ecological and behavioral cues present in the field, pre-zygotic reproductive isolation can be overcome in the laboratory, and hybridization can occur efficiently, with no observed post-zygotic incompatibility and even potential heterosis in some crosses. These findings differ from some laboratory studies reviewed in Nagoshi and Meagher [33], which reported varying degrees of mating incompatibilities between the strains, including reduced fertility in some interstrain crosses.

The observed discrepancies between studies may stem from environmental influences, as pheromone-mediated reproductive isolation tends to be more pronounced in natural populations [34]. Our findings demonstrate that interstrain mating can occur readily in the absence of ecological and behavioral pressures present in the field under these specific laboratory conditions. Additionally, genetic background differences between the tested populations in our study and those examined by Nagoshi and Meagher could explain the conflicting results, since certain strains may retain higher compatibility or even exhibit hybrid vigor [33]. These results indicate that the behavioral or ecological hybridization barriers observed in natural populations may not be observed under simplified laboratory conditions. This highlights the importance of interpreting laboratory hybridization results with caution and emphasizes the need for future research to clarify how environmental cues and genetic variation modulate reproductive isolation between these biotypes in nature.

Our findings, while based on limited strain collections from specific geographical origins, align with broader observations in the literature. Over evolutionary time, the host range of the CS appears to have narrowed, while the RS has maintained adaptability to a broader range of plants. Studies of fall armyworm populations in southern Florida found that RS populations dominate in environments with greater plant diversity and variability, such as wetlands and pastures [35,36]. Field and laboratory studies suggested RS had higher survival on two grasses being grown for biofuel production [37]. It should be noted, however, that our conclusions regarding the generalist nature of the RS and specialist characteristics of the CS are drawn from a limited set of host plants and laboratory conditions, and may not fully represent the ecological breadth of these strains in natural environments.

Analysis of fall armyworm samples from multiple regions has revealed that CS samples are rarely found on non-corn hosts [38], supporting the concept of higher host specificity in the CS. Our study provides experimental evidence consistent with these field observations, though we acknowledge that our relatively simple experimental design (testing only four hosts) limits comprehensive conclusions about host range breadth.

Notably, Acevedo et al. identified distinct salivary proteomes in the two strains [39,40], and genomic comparisons reveal RS-specific upregulation of detoxification enzymes, particularly in response to plant secondary metabolites [41]. While our hybridization experiments suggest that the enhanced detoxification capability in our RS colony is dominantly inherited, indicating this trait may be an acquired characteristic, further research with more diverse genetic material is needed to confirm the generalizability of this pattern.

Our reciprocal cross-experiments using these specific colonies suggest that the enhanced detoxification metabolism associated with the RS may be dominantly inherited. Similar heterosis effects, where F1 offspring exhibited enhanced fitness, have been observed in hybrids of other Lepidoptera, such as *Helicoverpa* species [42]. Potential mechanisms underlying this effect could include the overexpression of detoxification genes, key mutations, or gene duplication. These findings offer preliminary insights for future research into the genetic basis of detoxification mechanism. While the CS colony used in this study showed narrower host adaptability, it may possess high nutrient conversion efficiency on its host plants. The superior performance of offspring with a CS mother also hints at possible fitness advantages derived from the maternal CS lineage. Given that hybridization occurs under laboratory conditions, the potential ecological consequences, such as the transfer of adaptive traits in overlapping habitats, warrant further investigation under more naturalistic settings.

## 5. Conclusions

Our laboratory results confirm the absence of intrinsic post-zygotic barriers to hybridization between the CS and RS biotypes under controlled conditions. In this setting, efficient interstrain mating produces viable hybrids, and we observed that the RS’s enhanced detoxification metabolism and broader host adaptability were dominantly inherited in these hybrid offspring. While our findings occurred in a simplified environment lacking natural ecological and behavioral pressures, they raise the possibility that gene flow between biotypes could, under certain conditions, transfer adaptive traits. Therefore, further research is needed to assess the hybridization potential in the field and to understand the mechanisms behind these metabolic traits, which will be critical for evaluating long-term pest management strategies.

## Data Availability

All field trial raw data and other data not explicitly given in the manuscript, or figures are available upon request from the authors.
